# Amplitude da Onda Fibrilar: Devemos Usá-La Rotineiramente na Prática Clínica?

**DOI:** 10.36660/abc.20220680

**Published:** 2022-11-01

**Authors:** Andre Assis L. Carmo

**Affiliations:** 1 Hospital das Clinicas da Universidade Federal de Minas Gerais Belo Horizonte MG Brasil Hospital das Clinicas da Universidade Federal de Minas Gerais, Belo Horizonte, MG – Brasil

**Keywords:** Fibrilação Atrial, Frequência Cardíaca, Terapia por Estimulação Elétrica

A fibrilação atrial (FA) é a arritmia sustentada mais prevalente em todo o mundo e está associada a um aumento da morbidade e mortalidade em diferentes cenários clínicos, mesmo para pacientes com anticoagulação ideal e tratamento de controle do ritmo.^1,2^ Embora estudos anteriores não tenham mostrado o benefício da estratégia de controle do ritmo em comparação com o controle da frequência,^3,4^ dados recentes sugerem que as estratégias atuais de controle do ritmo, além do controle dos sintomas, podem melhorar os desfechos clínicos duros, incluindo mortalidade e acidente vascular cerebral.^5,6^

A estratégia de controle do ritmo refere-se às tentativas de restaurar ou manter o ritmo sinusal e inclui drogas antiarrítmicas, cardioversão elétrica e ablação de fibrilação atrial.^7^ Apesar da melhora progressiva nas estratégias de controle do ritmo ao longo dos anos, uma quantidade substancial de falhas na manutenção do ritmo sinusal,^8^ principalmente em pacientes com formas persistentes de FA, ressalta a importância de selecionar adequadamente os pacientes para estratégias de controle do ritmo.

Existem fatores bem conhecidos associados ao aumento do risco de recorrência de FA após a cardioversão, como idade avançada, sexo feminino, cardioversão prévia, doença pulmonar obstrutiva crônica, insuficiência renal, cardiopatia estrutural, maior índice de volume atrial esquerdo e insuficiência cardíaca.^1^ No entanto, considerando o impacto da FA sobre os pacientes e a economia da saúde, os esforços contínuos para melhorar a seleção do paciente para o controle do ritmo continuam sendo fundamentais no manejo da FA.

Nestes Arquivos Brasileiros de Cardiologia, Campelo et al.^9^ exploram a amplitude da onda fibrilatória como um preditor de cardioversão elétrica bem-sucedida e sua associação com múltiplos marcadores de eventos tromboembólicos aumentados. Avaliaram retrospectivamente 57 pacientes submetidos à cardioversão elétrica. A onda fibrilatória foi classificada de acordo com a amplitude na derivação V1. Onda fibrilatória ≥0,1 mV foi usada para definir FA de onda fibrilatória grossa.

A onda fibrilatória grossa não foi associada à presença de trombo atrial esquerdo nem ao contraste ecográfico espontâneo. A duração da FA, o volume do átrio esquerdo e a velocidade do fluxo do átrio esquerdo foram semelhantes em ambos os grupos.

Entretanto, o principal achado deste estudo foi a associação da amplitude da onda fibrilatória com o sucesso da cardioversão elétrica. A cardioversão elétrica foi realizada com aumento progressivo de energia, e o sucesso agudo foi definido se o ritmo sinusal foi mantido uma hora após o procedimento. Embora semelhante a muitos estudos, essa definição dificulta a obtenção de conclusões amplas sobre questões fisiopatológicas ou clínicas, uma vez que a recorrência imediata e a ausência de reversão ao ritmo sinusal provavelmente têm significados muito diferentes.

Na população de Campelo et al.,^9^ a onda fibrilatória grossa foi associada ao sucesso agudo da cardioversão (94,3% vs. 72,7%, p=0,036; OR 6,17; IC 95% 1,21-34,5). Além disso, as energias máxima e cumulativa foram maiores no grupo FA fina. Esses achados estão intimamente relacionados a outros estudos que mostram uma associação da FA fina ao aumento da prevalência de fibrose atrial esquerda,^10,11^ uma vez que a fibrose atrial deve ser um importante marcador de má resposta às terapias de controle do ritmo.

No que diz respeito à ablação de FA por cateter, a ferramenta mais eficaz para controle de ritmo,^12,13^ há estudos conflitantes correlacionando a amplitude da onda p fibrilatória ao sucesso da ablação da FA. Em 2009, Nault et al.^14^ avaliaram a associação da amplitude da onda fibrilatória com variáveis clínicas, ecocardiográficas e recorrência de FA em 90 pacientes submetidos à ablação de FA. Foi observada associação entre amplitude da onda F e recorrência de FA. Quarenta e três por cento dos pacientes com amplitude média da onda f <0,05 mV na derivação V1 tiveram recorrência de FA em comparação com 12% daqueles com onda F ≥0,05 mV (p = 0,004).

Mais recentemente, Squara et al.^10^ avaliaram a associação da amplitude da onda fibrilatória com a extensão das áreas de baixa voltagem em 29 pacientes submetidos à ablação por cateter para FA. A amplitude da onda fibrilatória correlacionou-se inversamente com a extensão das áreas de baixa voltagem do endocárdio atrial esquerdo. No entanto, a amplitude da onda fibrilatória não predisse a recorrência da FA após um seguimento de 23,3 ± 9,8 meses.

No contexto da cardioversão elétrica da FA, Zhao et al.^15^ avaliaram 94 pacientes estratificados pela amplitude da onda fibrilatória e determinaram as taxas de sucesso agudo (4 h após cardioversão) e médio prazo (6 semanas após cardioversão). Não houve diferença na taxa de sucesso agudo, energia máxima utilizada ou o número de choques necessários. Em 6 semanas, no grupo de onda fibrilatória grossa, 75% dos pacientes mantiveram SR vs. 40% no grupo de onda fibrilatória fina (p = 0,006). Apesar da diferença nas taxas de sucesso agudo, ambos os estudos mostram que as ondas fibrilatórias podem ter um papel na previsão de sucesso agudo ou de médio prazo na estratégia de controle do ritmo.

Resumindo, Campelo et al.^9^ fornecem mais evidências na amplitude da onda fibrilatória utilizada como marcador de melhor resposta na estratégia de controle do ritmo da FA. Mesmo com estudos conflitantes nesse cenário, há evidências substanciais de que a onda fibrilatória fina está associada à extensão da fibrose atrial e pode levar a resultados piores nas terapias de controle do ritmo. No entanto, mais estudos com um número maior de pacientes e avaliação de diferentes estratégias para controle do ritmo são necessários para serem utilizados rotineiramente na prática clínica.
