# Supplementary material for: Amplitude da Onda Fibrilar: Devemos Usá-La Rotineiramente na Prática Clínica?
Source: Arq Bras Cardiol. 2022 Nov 1;119(5):789–90. [Article in Portuguese] doi: 10.36660/abc.20220680 (PMC9750221; doi:10.36660/abc.20220680)
Supplement: Translation

# Translation: Fibrillatory Wave Amplitude: Should We Use It Routinely in Clinical Practice?

**Authors:** Andre Assis L. Carmo

**Affiliations:** 1 Belo Horizonte MG Brazil Hospital das Clinicas da Universidade Federal de Minas Gerais, Belo Horizonte, MG – Brazil

**Keywords:** Atrial Fibrillation, Electric Stimulation Therapy, Practice Guideline, Heart Rate

Atrial fibrillation (AF) is the most prevalent sustained arrhythmia worldwide and is associated with an increased burden of morbidity and mortality in different clinical scenarios, even for patients with optimal anticoagulation and rate control treatment.^1,2^ Although previous studies did not show the benefit of rhythm control strategy compared to rate-control,^3,4^ recent data suggest that current rhythm control strategies, in addition to symptom control, can improve hard clinical outcomes, including mortality and stroke.^5,6^

Rhythm control strategy refers to attempts to restore or maintain sinus rhythm and includes antiarrhythmic drugs, electrical cardioversion and atrial fibrillation ablation.^7^ Despite progressive improvement in rhythm control strategies over the years, a substantial amount of failure in maintaining sinus rhythm,^8^ mainly in patients with persistent forms of AF, underscores the importance of properly selecting patients for rhythm-control strategies.

There are well-known factors associated with increased risk of AF recurrence after cardioversion, such as older age, female sex, previous cardioversion, chronic obstructive pulmonary disease, renal impairment, structural heart disease, larger left atrial volume index, and heart failure.^1^ Nevertheless, considering AF's burden on patients and the health economy, continuous efforts to improve patient selection for rhythm control remain pivotal in AF management.

In this *Arquivos Brasileiros de Cardiologia*, Campelo et al.^9^ explore fibrillatory wave amplitude as a predictor of successful electrical cardioversion and its association with multiple markers of increased thromboembolic events. They retrospectively evaluated 57 patients who had undergone electrical cardioversion. The fibrillatory wave was classified according to amplitude in lead V1. Fibrillatory wave ≥0,1 mV was used to define coarse fibrillatory wave AF.

The coarse fibrillatory wave was not associated with the presence of left atrial thrombus nor spontaneous echo contrast. AF duration left atrial volume and left atrial flow velocity were similar in both groups.

Notwithstanding, the main finding of this study was the association of fibrillatory wave amplitude to electrical cardioversion success. Electrical cardioversion was performed with progressive energy increase, and acute success was defined if sinus rhythm was maintained one hour post-procedure. Although similar to many studies, this definition makes it difficult to draw broad conclusions concerning pathophysiological or clinical issues, given that immediate recurrence and absence of reversion to sinus rhythm probably have very different meanings.

In Campelo et al.^9^ population, coarse fibrillatory wave was associated to acute success cardioversion (94,3% vs. 72,7%, p=0,036; OR 6,17; IC 95% 1,21-34,5). In addition, maximum and cumulative energies were higher in the fine AF group. These findings are closely related to other studies showing an association of fine AF to increased prevalence of left atrial fibrosis,^10,11^ since atrial fibrosis is supposed to be an important marker of poor response to rhythm control therapies.

Concerning catheter-based ablation of AF, the most effective tool for rhythm control therapies,^12,13^ there are conflicting studies correlating fibrillatory p wave amplitude to AF ablation success. In 2009, Nault et al.^14^ assessed the association of fibrillatory wave amplitude with clinical, echocardiographic variables and AF recurrence in 90 patients who underwent AF ablation. An association between F-wave amplitude and AF recurrence was observed. Forty-three percent of patients with mean f wave amplitude <0.05 mV in lead V1 had AF recurrence compared to 12% of those with F-wave ≥0.05 mV (p = 0.004).

More recently, Squara et al.^10^ evaluated the association of fibrillatory wave amplitude to the extent of low voltage areas in 29 patients who underwent catheter ablation for AF. The fibrillatory wave amplitude was inversely correlated to the extent of left atrial endocardial low-voltage areas. However, fibrillatory wave amplitude did not predict AF recurrence after a follow-up of 23.3 ± 9.8 months.

In the context of electrical cardioversion of AF, Zhao et al.^15^ evaluated 94 patients stratified by fibrillatory wave amplitude and determined acute (4 h after cardioversion) and medium-term (6 weeks after cardioversion) success rates. There was no difference in acute success rate, maximal energy used or the number of shocks required. At 6 weeks, in the coarse fibrillatory wave group, 75% of patients maintained SR vs. 40% in the fine fibrillatory wave group (p = 0.006). Despite the difference in acute success rates, both studies show that fibrillatory waves can have a role in predicting acute or medium-term success in the rhythm control strategy.

In summary, Campelo et al.^9^ provide further evidence in fibrillatory wave amplitude used as a marker of better response in rhythm control strategy of AF. Even with conflicting studies in this scenario, there is substantial evidence that fine fibrillatory wave is associated with the extent of atrial fibrosis and can lead to poorer results in rhythm control therapies. However, further studies with a larger number of patients and evaluation of different strategies for rhythm control are required to be routinely used in clinical practice.
